# Shift of Sensitivity in *Botrytis cinerea* to Benzimidazole Fungicides in Strawberry Greenhouse Ascribing to the Rising-lowering of E198A Subpopulation and its Visual, On-site Monitoring by Loop-mediated Isothermal Amplification

**DOI:** 10.1038/s41598-019-48264-4

**Published:** 2019-08-12

**Authors:** Y. H. Liu, S. K. Yuan, X. R. Hu, C. Q. Zhang

**Affiliations:** 1 Department of Plant Pathology, Zhejiang Agriculture and Forest University, Lin’an, 311300 China; 2 Institute for the Control of Agrochemicals, Ministry of Agriculture, Beijing, 100026 China

**Keywords:** Pathogens, Fungal pathogenesis

## Abstract

Grey mold disease results from *Botrytis cinerea*, a classical “high-risk” plant pathogenic fungus in meaning of resistance development to fungicides, and its management depends largely on the frequent applications of fungicides. The evolution of resistance to benzimidazole chemicals during 2008 and 2016 was monitored continuously in strawberry greenhouses located in Zhejiang province. Results showed that extensive applications of the mixture of carbendazim and diethofencarb caused the rapid spread of Ben MR subpopulation. The withdraw of this mixture lead to the sharply decrease of Ben MR and re-dominance of Ben HR isolates of *B*. *cinerea* with the E198A mutation in *β-tubulin* gene. The LAMP primers, based on the E198A point mutation, were designed to detect the E198A genotype specifically. HNB (Hydroxynaphthol blue), a metalion indicator, acted as a visual LAMP reaction indicator that turned the violet colored into a sky-blue color. The detection limit of concentration of DNA was 100 × 10^−2^ ng/μL and this LAMP assay could be applied to detect the E198A genotype with 100% accuracy in strawberry greenhouses of three Province and was more rapid and easier to operate. In summary, we establish a simple and sensitive on-field LAMP assay which can be adopted to determine within 1.5 h whether the benzimidazoles or the mixture of a benzimidazole fungicide and diethofencarb is suitable for management of *B*. *cinerea*.

## Introduction

*Botrytis cinerea*, the causative agent of grey mold disease, is an omnipresent plant pathogenic fungus distributed worldwide^1^. It affects more than 1000 species of 586 plant genera native to most continents^2,3^ and infects all parts of plants including seeds, seedlings, fruits, leaves, flowers, at pre-harvest and post-harvest stages such as storage, the process of transport or during the period of retail and display^4^. In general, grey mold results in 20 to 30% yield loss and even more than 50% when the environment is favorable for *B*. *cinerea* epidemic^5,6^. On strawberry (*Fragaria* × *ananassa*), for example, one of the most important fruit crops in China and worldwide, this disease will seriously occur under moist weather with the temperatures between 20 and 23 °C. The primary source of inoculum is conidia which usually spread by air and water, and may infect strawberry plants especially via flowers, but also surface injured fruits^2^.

Management of grey mold disease is achieved by frequent applications of fungicides. However, *B*. *cinerea* is a typical “high-risk” pathogenic fungus. Under the selection pressure of a fungicide, once the resistance occurs, the level of resistance will develop rapidly for its short life cycle and prolific reproduction rate^7^. The failure of control due to the rapid evolution of resistance has become a important concern. Benzimidazole fungicides, the first group of chemicals with therapeutic activity were one of the main agents used in crop plant productions^8–10^. The phenomena that fungi resistant to benzimidazoles has been demonstrated by a number of studies in different plant diseases^11–13^. *B*. *cinerea* resistant to benzimidazoles has been reported to carry point mutations at the codon 198 (E198A or E198K or E198V) or 200 (F200Y) in the *β-tubulin* gene^14–16^. In general, two types of benzimidazole-resistants, Ben HR (high resistance to benzimidazole fungicides) and Ben MR (moderately resistant to benzimidazoles), were widely detected in fields. Ben HR isolates were caused by the E198A mutation which simultaneously showed more sensitive to diethofencarb, a phenylcarbamate, than the wild benzimidazole-sensitive (Ben S) isolates. Ben MR isolates, simultaneously resistant to diethofencarb just like the Ben S isolates and caused by the F200Y mutation, were detected after the applications of the mixture “a benzimidazole fungicide + diethofencarb^14,15,17^”.

Resistance to benzimidazoles in plant-pathogenic fungi such as *B*. *cinerea* is traditionally detected by the tests of discriminatory doses, which takes 3 to 5 days even extended to a week, and is time-consuming and laborious^10,18,19^. Several detection methods based on PCR including AS-PCR and RAPD-PCR have been applied for the detection of mutant isolates responsible for fungicide resistance^9,20^. Previous studies have developed PCR-RFLP and real-time PCR to detect benzimidazole-resistance with mutations at codon 198 in the β-tubulin gene in *B*. *cinerea* and *Monilinia laxa*^9,21^. However, these techniques have inherent shortcomings, including the need for prolonged time and expensive equipment, which limit these methods to laboratories and are not suitable for field assays. Loop-mediated isothermal amplification (LAMP)^22^ is a relatively new and rapid nucleic acids amplification technique which does not need a precision thermal cycler, thus making it appropriate for using in the fields. The LAMP assay has been used to detect several plant-pathogenic fungi, including *Magnaporthe grisea*^23^, *Erysiphe necator*^24^, *Phytophthora sojae*^25^, and others^26–28^. The current study is conducted to (i) monitor the evolution of resistance in *B*. *cinerea* from strawberry greenhouse following different applications of benzimidazoles, (ii) develop a simple, rapid and sensitive on-field LAMP assay for E198A subpopulation to guide the applications of benzimidazole fungicides for management of *B*. *cinerea*.

## Results

### Shift of sensitivity to benzimidazoles during 2008 and 2016

**Figure 1:**
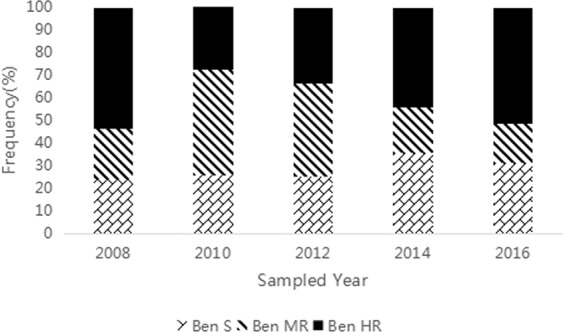
Evolution of resistance to benzimidazoles in strawberry greenhouses during 2008 and 2016. Ben HR = Highly resistant to benzimidazole fungicides, Ben MR = Moderately resistant to benzimidazole fungicides, Ben S = Sensitive to benzimidazole fungicides.

The kind of chemicals used showed great impact on the evolution of sensitivity in *B*. *cinearea* to benzimidazoles (Fig. 1). In these tested 27 greenhouses located in Zhejiang province, the mixture of carbendazim and diethofencarb was adopted to control the Ben HR isolates since 2006, the rapid increase of Ben MR isolates (22.2%, 47.0%) and simultaneous decrease of Ben HR isolates (53.3%, 27.3%) were observed respectively for 2008 and 2010. After this mixture was not used in these greenhouses since 2011, Ben MR isolates decreased rapidly according to the frequency of 41.0%, 20.3%, and 17.6%, respectively for 2012,2014, and 2016. Meanwhile, re-increase of Ben HR subpopulation was observed by the frequency of 33.8%, 43.8%, and 51.1%. Moreover, the analysis of the *β-tubulin* gene fragments of the twenty isolates of different phenotype of benzimidazole sensitivity chosen at random per sampled year indicated all the Ben HR isolates had the E198A mutation and all Ben MR had the F200Y mutation.

### LAMP primers for detection of E198A *B*. *cinerea*

**Figure 2:**
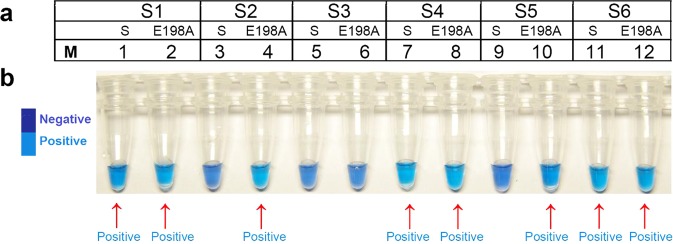
Determining six sets (S1–S6) of loop-mediated isothermal amplification (LAMP) primers. Label 1, 3, 5, 7, 9, 11: the template DNA was extracted from the carbendazim sensitive strain (S). Label 2, 4, 6, 8, 10, 12: the template DNA was extracted from the isolates of *Botrytis cinerea* with the E198A mutation (E198A). (**a**) LAMP primers sets. (**b**) HNB-based visual changes in colors. The positive samples were pointed out by red arrows.

Template DNA from CL-3, a Ben HR isolate carrying the E198A mutation, and a Ben S isolate (TM-10) were used to assess the six sets of mismatched LAMP primers (FIP was mismatched) both visually based on color changes in Hydroxynaphthol blue (HNB) (Fig. 2b). The results indicated that the different FIP primer sets S2 (F3/B3/FIP2/BIP) and S5 (F3/B3/FIP5/BIP) could be used to distinguish the E198A genotype of *B*. *cinerea* (Fig. 2 and Table 1). The primer set S2 showed the most intensive sky blue color which distinguish the E198A isolates from Ben S isolates with ease and therefore was adopted in subsequent tests in this study.

**Table 1:** Primers used to develop the LAMP assay for rapid detection of carbendazim-resistant *Botrytis cinerea* with E198A mutation in β-tubulin gene.

Primer name	Primer set name	Type	Sequence (5′-3′)
BC-198-F3		Forward outer	CAACTCTCTCTGTCCATCAA
BC-198-B3		Reverse outer	GGAGATCTGAGTTAAGTTGACC
BC-198-FIP1	S1	Forward inner	TCTCATGCAAATATCGTAAAGAGCCTGGTTGAGAACTCTGACGC
BC-198-FIP2	S2	Forward inner	TCTCATGCAAATATCGTAAAGAGCCTGGTTGAGAACTCTGAC**C**C
BC-198-FIP3	S3	Forward inner	TCTCATGCAAATATCGTAAAGAGCCTGGTTGAGAACTCTGAC**C**CG
BC-198-FIP4	S4	Forward inner	TCTCATGCAAATATCGTAAAGAGCCTGGTTGAGAACTCTGAC**T**C
BC-198-FIP5	S5	Forward inner	TCTCATGCAAATATCGTAAAGAGCCTGGTTGAGAACTCTGAC**A**C
BC-198-FIP6	S6	Forward inner	TCTCATGCAAATATCGTAAAGAGCCTGGTTGAGAACTCTGA**AG**C
BC-198-BIP		Reverse inner	CAGCAACCCATCTTACGGAGAAAACGGAGACAGGTGGTA
BCtubF		Forward	AGGTACCATGGATGCTGTCC
BCtubR		Reverse	AAATGGCAGAGCATGTCAA

### Sensitivity of LAMP in the laboratory

**Figure 3:**
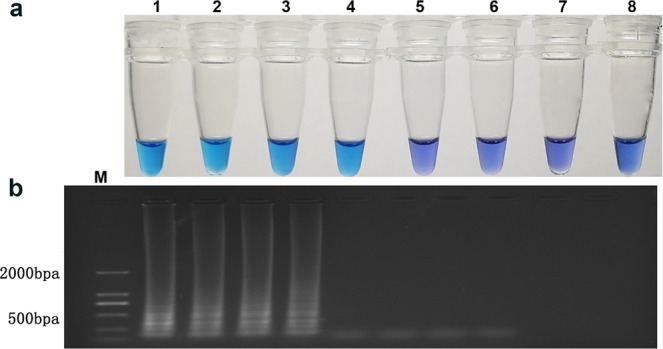
Sensitivity test of LAMP to detect the field diseased strawberry fruits. Reaction tubes 1–7 were 10-fold dilutions of the cleavage products, with DNA concentrations of 100, 100 × 10^−1^, 100 × 10^−2^, 100 × 10^−3^, 100 × 10^−4^, 100 × 10^−5^, 100 × 10^−6^ ng/μL, and the tube 8 was ddH_2_O blank control. (**a**) Sensitivity detection on the basis of HNB color change. (**b**) Sensitivity detection on the basis of gel electrophoresis detection.

For the sensitivity of the LAMP tests, 10-fold diluted cleavage products were used as DNA templates for determining the sensitivity based on the visible color change indicating by HNB in the tube (Fig. 3a) and the results from gel electrophoresis (Fig. 3b). The detection limit of the LAMP assay of DNA was 100 × 10^−2^ ng/μL.

### Specificity and repeatability of the LAMP assay

**Figure 4:**
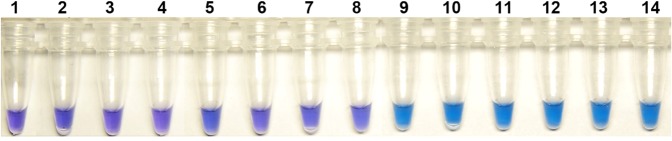
Specificity and accuracy of the developed LAMP assay to detect different isolates of *Botrytis cinerea*. Label 1 to 8: wild-type, E198V, E198K, wild-type, F200Y, E198V, E198K, wild-type, respectively. Label 9 to 14: Isolates with E198A mutation.

The LAMP assay was positive only for all 6 isolates harboring the E198A mutation. No positive DNA products were observed when other type of mutants or Ben S isolates were used as templates (Fig. 4). These results suggested that the established LAMP assay had good specificity, accuracy and stability.

### On-site LAMP detection and traditional tests in laboratory

To evaluate the reliability and significance in fields, we adopted samples from different greenhouses in five different geographical regions located in three different Province by using the on-site LAMP assays. Results indicated that 153 (67.4%) samples were positive reactions. In laboratory, the traditional tests by the discriminatory dose of 5 μg mL^−1^ showed that 169 out of 227 isolates (74.4%) were resistant (Ben R) which could grow on PDA plates amended with 5 μg mL^−1^ carbendazim and the remaining 58 isolates were sensitive (Ben S). Further mutation analysis by PCR showed that a total of 169 isolates had mutant genotypes of *β-tubulin* gene (153 with E198A, 5 with E198V, 4 with E198K, and 7 with F200Y) and 58 were Ben S isolates. Thus, the on-site LAMP assay could specifically detect all isolates with the E198A mutation with 100% accuracy in fields (Table 2).

**Table 2:** Specificity and accuracy of the developed LAMP assay on-site detecting *Botrytis cinerea* from different geographical origins.

Origin	Number of samples	LAMP Positive	Traditional test Positive^x^	Number of different mutation genotypes			
				E198A	E198V	E198K	F200Y
Bozhou, Anhui	28	8	8	8	0	0	0
Lin’an, Zhejiang	43	33	38	33	3	0	2
Changle, Zhejiang	84	77	80	77	0	0	3
Shaoxing, Zhejiang	37	16	21	16	2	3	0
Shijiazhuang, Hebei	35	19	22	19	0	1	2
Total	227	153	169	153	5	4	7

^X^Traditional test positive mean this isolate could grow on PDA plates amended with 5 μg ml^−1^ carbendazim.

## Discussion

As a high risk pathogen, resistance of *B*. *cinerea* to fungicides can emerge rapidly after continuous exposures in the fields^29^. Once resistance occurs, the control efficiency reduces sharply in addition to the increased fungicide residues threatening the health of humans as well as other non-target organisms. At present, there is a need for environment-friendly and sustainable control measures that requires smarter usages of fungicides in order to delay or manage resistance^1^. Therefore, monitoring the evolution of fungicide resistance is an important requisite for integrated control of grey mold.

In China, the selection of resistance in *B*. *cinerea* from crops such as vegetables to benzimidazoles and double-resistance to benzimidazoles and diethofencarb has been reported in regions such as Zhejiang^16,17,30^. In this study, the development of resistance to benzimidazoles was monitored during 2008 and 2016 in strawberry greenhouses of Zhejiang Province. Results showed that the application strategy of fungicides had significant impact on the evolution of sensitivity. The extensive usages of the mixture of carbendazim and diethofencarb resulted in quick increase of Ben MR subpopulation. Interestingly, when its applications were withdrawn for serious double-resistance to benzimidazoles and diethofencarb, Ben HR isolates re-dominated in *B*. *cinerea* population although no selection pressure of benzimidazole fungicides existed in the latter process. And, all the Ben HR isolates detected after that re-dominance had the E198A (GAG → GCG) mutation in *β-tubulin* gene. This evolution phenomenon of sensitivity might be mainly attributed to their strong competitive ability of Ben HR isolates^1^. Many studies found that E198A was the dominant sub-population with resistance to benzimidazole fungicides^16,29^. In 2017, we tested samples from three Province in China, and the results showed that 81.9% resistant isolates had the single point mutation, E198A, and only a frequency of 3.1% was observed for F200Y. A total of frequency of 4.0% was detected for other two genotypes (E198V and E198K) of benzimidazole resistance. However, E198V, for example, was reported as lowly resistant (LR) which can grow on 5 mg L^−1^ but cannot on 10 mg L^−1^ carbendazim or thiophanate-methyl and were more sensitive to low temperature according to our previous study^16^. Therefore, monitor the E198A sub-population can give enough information for the shift of sensitivity and management of grey mold.

Previous studies also indicated that the frequency of sub-populations harboring the F200Y mutation in *B*. *cinerea* decreased rapidly if application of carbendazim and diethofencarb mixture was stopped^29^. We speculated that this evolution pattern of resistance would be utilized to control *B*. *cinerea* through a well-directed application of benzimidazoles if we could determine the instantaneous status of population structure of *B*. *cinerea* in a greenhouse. When E198A subpopulation dominates, the mixture of carbendazim and diethofencarb can be used. The decrease of E198A isolates and rapid development of Ben MR will be expected due to extensive applications of the mixture. Then, the mixture and the benzimidazole fungicides should be suspended for some times until the first situation appeared which would happen within a shorter period. This suggests that we can get enough information for the management of grey mold through monitoring the situation of E198A sub-population. However, the prerequisite is that we can rapidly determine the status of E198A subpopulation.

The discriminatory doses assay is the commonly used method to detect resistance to benzimidazole fungicides^10,16,31^. However, it is not suitable for field applications where quick detection is preferred. Molecular-based methods such as Real-time PCRs^9,20,21^ not only require 4 to 5 h but also expensive equipment and well-trained technicians that limit their applications in fields. In this study, the E198A mutants of *B*. *cinerea* were detected by a novel LAMP assay which could successfully differentiate E198A mutants from sensitive isolates and other mutants (E198V, E198K, and F200Y) with 100% accuracy. For sample pre-treatments, using All-DNA-Fast-Out to extract DNA within about 10 min, eliminated the process of centrifugation, extraction and other operational steps in the traditional procedure of extracting DNA, thereby saving time of the samples-pretreatment, and reducing the contamination between the samples, to achieve the purpose of rapid detection. This assay could be on-field finished within 1.5 h without expensive devices thus provided an alternative approach to rapidly detect fungicide resistance in the plant pathogens in fields. LAMP is recently developed for detection of plant pathogenic fungi in infected plant materials^32,33^. Addition of the HNB dye prior to amplification, which is a metal ion that is widely used in LAMP assays, reduces the risk of contamination^34,35^. In this study, the negative and positive responses were successfully distinguished by HNB color changes^30,36,37^. The LAMP detection for *B*. *cinerea* based on *Bcos5* and DNA was extracted using the Plant Genomic DNA Kit was 10-fold more sensitive than conventional PCR^32^. They also reported a specific, repeatable and sensitive LAMP assay for detection of *B*. *cinerea* with F200Y mutation (Ben MR) from different plants artificially inoculated with conidia^30^. Our study described a LAMP assay targeting E198A mutation of *B*. *cinerea* (Ben HR) according the evolution pattern of different sensitivity to fungicides in strawberry greenhouses to guide the application of benzimidazole fungicides. Moreover, as *Botrytis cinerea* is a ubiquitous and “high-risk“of fungicide resistance development plant pathogenic fungus worldwide, the smarter and precise usages of fungicides on plants through on-site monitoring techniques will significantly decrease the input of fungicides and therefore provide benefits for food security and human healthy.

## Materials and Methods

### Fungicides

Technical grade carbendazim (98% a.i.) provided by the Institute for the Control of Agrochemicals, Ministry of Agriculture (CAMA), P.R. China was dissolved in 0.1 mol/L hydrochloric acid (HCl) to prepare the stock solutions which were stored at 4 °C in the dark. The stock solutions were added to molten media, when they were cooled to approximately 50 °C.

### Monitor the evolution of sensitivity to benzimidazoles during 2008 and 2016

The total of 135, 132, 139, 128 and 131 single-spored isolates were recovered continuously from 27 strawberry greenhouse in Zhejiang Province in 2008, 2010, 2012, 2014 and 2016, respectively, as the reference described^38^. In these sampled greenhouses, benzimidazole fungicides, including carbendazim and thiophanate-methyl, were frequently used before 2004 and their application decreased rapidly, which were replaced by the mixture of carbendazim and diethofencarb (an *N*-phenylcarbamate) since 2006. From 2011, this mixture was not used in these greenhouses due to poor efficacy. Resistance to benzimidazoles of the collected isolates was determined through the tests of discriminatory doses according to previous described^16^. In brief, isolates which could not grow on potato dextrose agar (PDA) (200 g potato, 20 g dextrose, 35 g agar and 1 L H_2_O) plates amended with 5 mg/L carbendazim were considered as sensitive (Ben S); those could grow on 10 mg/L but not on 100 mg/L were defined as moderately resistance (Ben MR); and those that could grow on 100 mg/L were determined as highly resistant (Ben HR).

### Isolation the *β-tubulin* gene fragments of *B*. *cinerea*

Twenty isolates of different phenotype of benzimidazole sensitivity for each sampled year were chosen at random. The DNA fragment, which included the 198^th^ and 200^th^ codon of the *β-tubulin* gene, was amplified by conventional PCR using BCtubF and BCtubR (Table 1). The volume of the reaction was 50 μL with the reagents as following: 25 μL 2 × PCR Master, 0.4 μM primers, 1 μL DNA template, ddH_2_O was supplemented to 50 μL and the thermal cycling of conventional PCR program was 95 °C for 5 min; 30 reaction cycles of 95 °C for 30 s, 55 °C for 30 s, and 72 °C for 90 s with an extension at 72 °C for 5 min. The PCR products were analyzed through gel electrophoresis and purified using UNIQ-10 Coloum DNA Purification Kit (Sangon, Shanghai), following the manufacturer’s protocol. All the PCR products were sequenced by Invitrogen Company, Shanghai, China. The sequences were aligned using Crustal W software (http://www.bi.ac.uk).

### Primer design and evaluation for LAMP detection of E198A *B*. *cinerea*

The Primer explorer V5 software tools (http://primerexplorer.jp/e/) were used to design LAMP primers which targeted a fragment containing the codon 198^th^ and 200^th^ in the β-tubulin gene of *B*. *cinerea* (FQ790278.1). The detailed structure of LAMP primers used is shown in Fig. S1. Mismatching bases were introduced at the 3′end of the FIP, and six groups of specific LAMP primers (Table 1) were designed to differentiate E198A mutants from the wild-type. Two *B*. *cinerea* isolates, CL-3 (E198A, Ben HR) and TM-10 (Ben S) were used to develop the LAMP assay. The six groups of mismatched LAMP primes were screened to determine their specificity to distinguish the E198A genotype from the sensitive type. The reaction volume was a 25 μL mixture: 8 U *Bst* DNA polymerase (New England Biolabs, Beijing), 1 mM dNTPs (Sangon, shanghai), 4 mM Mg^2+^, 0.8 M betaine (Sigma), 1.2 μM FIP and BIP, 0.2 μM F3 and B3, 150 μM HNB (metalion indicator, Sigma), 2.5 μL 10 × Thermo Pol buffer, 1 μL double-stranded target DNA (a concentration of 500 ng/μL). The LAMP reaction mixture was performed in 0.2 mL microcentrifuge tubes with the genomic DNA of CL-3 as the positive control template, DNA of TM-10 and ddH_2_O as negative control. To enable visualization, HNB was added to the reaction mixture. After optimization, reactions were run at 65 °C for 60 min. The LAMP amplification product was visually observed in daylight. If the color of HNB in reaction tubes turned from violet to sky blue, it was considered as positive, while a violet color of the HNB in reaction mixture indicated a negative reaction. Each treatment was set up in triplicate and the tests were repeated at least three times.

### Analyzing the sensitivity of LAMP in the laboratory

The isolate CL-3 was cultured at 22 °C for 3 days on PDA plates and mold was picked from the colony surface to extract DNA with All-DNA-Fast-Out (Sangon, Shanghai), according to the manufacturer’s protocol. After that, tubes were heated at 80 °C for 10 min in a heated block. The supernatant obtained were quantified by spectrophotometry (DNA concentration: 100~150 ng/μL). Then the lysate was 10-fold diluted in All-DNA-Fast-Out and used as DNA templates for testing the LAMP sensitivity. The final concentrations of DNA templates were 100, 100 × 10^−1^, 100 × 10^−2^, 100 × 10^−3^, 100 × 10^−4^, 100 × 10^−5^, and 100 × 10^−6^ ng/μL. The lowest DNA concentration at which positive results were observed represented the LAMP detection limit. Reaction results were observed by HNB color change. Each treatment was set up in triplicate and the tests were repeated at least three times.

### Determination of specificity and accuracy for LAMP tests

The specificity and accuracy of LAMP was verified by performing the assay using DNA of the wild type isolate, E198A mutants (n = 6) and other carbendazim-resistant mutants of *B*. *cinerea*. The LAMP assay was performed and assessed as described in the previous section. Each treatment was set up in triplicate and the tests were repeated at least three times.

### Comparison of on-site LAMP detection and traditional tests in laboratory

For the purpose of assessing this LAMP for on - site detection, a total of 227 grey mold diseased strawberry fruits (Table 2) from greenhouses chosen at random of five different geographical regions in Anhui, Zhejiang and Hebei Province were tested on 2017. For each fruit, approximately 2 mg mold was picked out by inoculation needle from the fruit surface and directly added to 0.2 mL microcentrifuge tubes containing 50 μL of All-DNA-Fast-Out (Sangon, Shanghai), after that, tubes were heated at 80 °C for 10 min in a heated block, the supernatant was directly used for LAMP assay as described above. Meanwhile, each tested strawberry fruit was respectively taken back to the laboratory to be tested with the the tests of discriminatory doses as described above. To confirm the results, the DNA fragment, which included the 198^th^ and 200^th^ codon of the *β-tubulin* gene, was amplified by conventional PCR as described above for each isolate collected on 2017.

## Supplementary information

Fig S1
