# Supplementary material for: Shift of Sensitivity in Botrytis cinerea to Benzimidazole Fungicides in Strawberry Greenhouse Ascribing to the Rising-lowering of E198A Subpopulation and its Visual, On-site Monitoring by Loop-mediated Isothermal Amplification
Source: Sci Rep. 2019 Aug 12;9:11644. doi: 10.1038/s41598-019-48264-4 (PMC6690993; doi:10.1038/s41598-019-48264-4)
Supplement: Supplementary file 1 — Fig S1 [file 41598_2019_48264_MOESM1_ESM.docx]

**Shift of Sensitivity in *Botrytis cinerea* to Benzimidazole Fungicides in Strawberry Greenhouse Ascribing to the Rising-lowering of E198A Subpopulation and its Visual, On-site Monitoring by Loop-mediated Isothermal Amplification**

**Y. H. Liu ^1^, S. K. Yuan^2^, X. R. Hu ^1^, and C. Q. Zhang^1^**

^1^ Department of Plant Pathology, Zhejiang Agriculture and Forest University, Lin’an 311300, China, ^2^Institute for the Control of Agrochemicals, Ministry of Agriculture, Beijing 100026, China;

**Correspondence**

(🖂) C. Q. Zhang, Department of Plant Pathology, Zhejiang Agriculture and Forest University, Wusujie 666, Hangzhou 311300, China

E-mail: cqzhang@zafu.edu.cn; Tel.: +086 -57163743089.


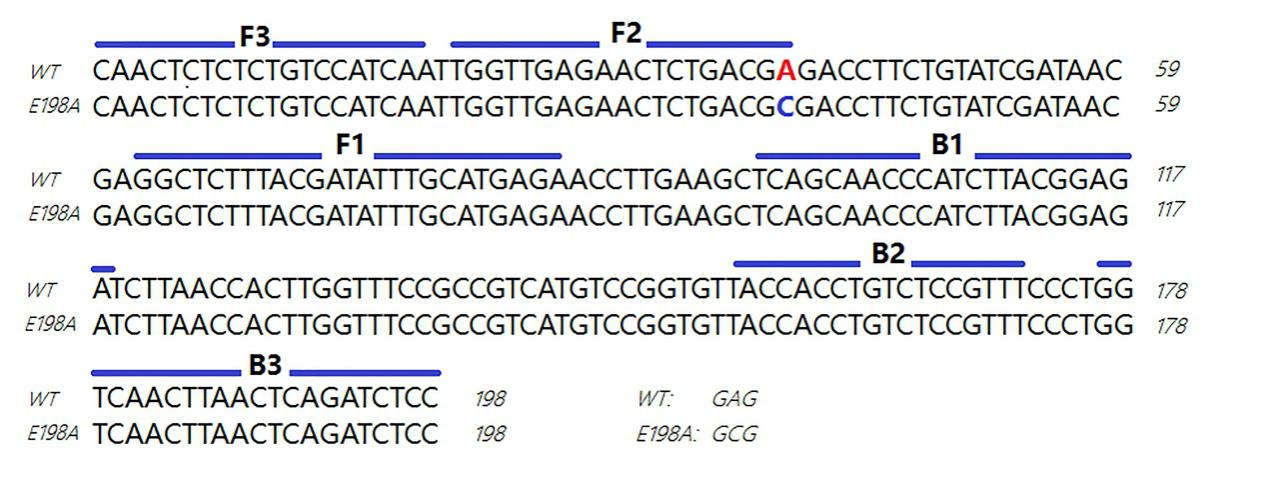


**Figure S1** Location of LAMP primers in the β-tubulin gene of *Botrytis cinerea* used to detect the E198A mutant genotype*.* Bold lines indicate location of the primers
